# Continuous time Bayesian networks identify Prdm1 as a negative regulator of TH17 cell differentiation in humans

**DOI:** 10.1038/srep23128

**Published:** 2016-03-15

**Authors:** Enzo Acerbi, Elena Viganò, Michael Poidinger, Alessandra Mortellaro, Teresa Zelante, Fabio Stella

**Affiliations:** 1 Singapore Centre on Environmental Life Sciences Engineering (Nanyang Technological University), Singapore 637551; 2 Singapore Immunology Network (SIgN), A^*^STAR, 8A Biomedical Grove, Immunos #04-06, Singapore 138648; 3 Department of Experimental Medicine, University of Perugia, 06132 Perugia, Italy; 4 Department of Informatics, Systems and Communication, University of Milano-Bicocca, Viale Sarca 336, Building U14, 20126 Milan, Italy

## Abstract

T helper 17 (TH17) cells represent a pivotal adaptive cell subset involved in multiple immune disorders in mammalian species. Deciphering the molecular interactions regulating TH17 cell differentiation is particularly critical for novel drug target discovery designed to control maladaptive inflammatory conditions. Using continuous time Bayesian networks over a time-course gene expression dataset, we inferred the global regulatory network controlling TH17 differentiation. From the network, we identified the *Prdm1* gene encoding the B lymphocyte-induced maturation protein 1 as a crucial negative regulator of human TH17 cell differentiation. The results have been validated by perturbing *Prdm1* expression on freshly isolated CD4^+^ naïve T cells: reduction of *Prdm1* expression leads to augmentation of IL-17 release. These data unravel a possible novel target to control TH17 polarization in inflammatory disorders. Furthermore, this study represents the first *in vitro* validation of continuous time Bayesian networks as gene network reconstruction method and as hypothesis generation tool for wet-lab biological experiments.

Interleukin-17 (IL-17) is an inflammatory cytokine produced by different immune cell types^1^. In particular, a T lymphocyte subset, termed TH17, is recognized as the main IL-17 producer in mammalian species. The main function of TH17 cell is the recruitment of inflammatory immune cells into the infected or damaged tissue during an inflammatory response. This inflammatory environment can lead to the exacerbation of autoimmune diseases, as well as chronic inflammatory conditions^2^. Still debated is the role of TH17 cells during infection, as there is evidence that elevated IL-17 levels exacerbate the disease outcome. Therefore, the amount of IL-17 released in those conditions is particularly relevant since elevated IL-17 levels may play a pathologic role in inflammatory and autoimmune diseases^3^. For this reason, many studies have focused on understanding the mechanisms responsible for TH17 differentiation^4^. Differentiation of TH17 cells is known to be triggered by cytokines, such as TGF-β, IL-6, and IL-1β^5^, and to depend on the transcription factors RORγt and RORα, required for the transcription of the *Il17* gene. However, RORγt is not sufficient to describe the full TH17 program of differentiation. Indeed, RORγt controls the final steps of the entire differentiation regulating the IL-23 receptor and the chemokine receptor CCR6 expression^6^,^7^.

Importantly, multiple transcription factors, including BATF and IRF4 are required for induction of RORγt^8^,^9^. Mainly described in murine cells are also the transcription factor c-Maf, Runx1, and Ahr^10^,^11^. Previously, Ciofani *et al*.^12^ used a number of data integration approaches to combine ChIP-seq, RNA-seq and microarray data with the aim of delineating the TH17 global transcriptional regulatory network on murine cells. The authors found that BATF and IRF4 contribute to chromatin accessibility and, with STAT3, trigger a transcriptional program characterizing the TH17 development. Unexpectedly, they also discovered negative regulators such as c-Maf as being able to attenuate the expression of pro-inflammatory loci. Despite the many studies, the network and regulation of those transcription factors that initiate and drive the development of TH17 cells remains unknown. Thus, investigating the regulatory network that controls the TH17 cell differentiation and the production of IL-17 is of utmost importance^2^.

The task of uncovering the causal structure of regulatory interactions (often referred to as “*gene network reconstruction*”) represents an open challenge in computational biology^13^,^14^. The increasing availability of high granularity time-course gene expression data offers an opportunity for in-depth study of the dynamic evolution of gene interaction networks. However, most of state-of-the-art network reconstruction approaches, which are exhaustively reviewed in refs 15, 16, 17, 18, have been conceived before the advent of omics technologies. Thus, such methods are not always able to fully exploit the dynamic nature of the data. Continuous time Bayesian networks (CTBNs)^19^ overcome such limitation by implementing a continuous representation of the time. In a CTBN variables are free to evolve continuously over time as a direct function of a continuous time conditional Markov process, and the factored state representation, which keeps the computation feasible, derives from the theory of Bayesian networks. CTBNs present several advantages which make them suitable for gene network reconstruction tasks. The structural learning for CTBNs can be solved in polynomial time with respect to the dimension of the dataset. Furthermore, CTBNs can effectively model variables evolving at different time granularities or datasets characterized by measurements unevenly distributed over time (not equally spaced in time). The absence of assumptions on the temporal dynamics of the systems makes the inference independent of the data sampling intervals, thus improving model expressiveness^20^. CTBNs have been recently applied for the first time to the analysis of molecular data to investigate the regulatory interactions that characterize pathogenic versus non-pathogenic murine TH17 cells^21^. CTBNs have been proved to be more effective than other state-of-the-art methods at reconstructing regulatory interactions from time-course expression data^21^.

The graphical component of a CTBN provides the biologist with an intuitive level of abstraction of how the regulatory process take place over the duration of the experiment. For instance, a transcription factor that needs to be persistently activated during the whole duration of the process will likely be at the top of the inferred network hierarchy and characterized by a moderate/high degree of outgoing arcs. Similarly, transcription factors which are activated in a later phase, or that exert their function only limited to specific time intervals, will appear at an intermediate level of the network hierarchy and will be characterized by having both incoming and outgoing connections. Intuitively, genes that do not have influence on other genes (i.e. cytokines) will appear as being leaf nodes and characterized by having incoming arcs only. It is important to notice that a CTBN does not merely encodes the temporal order at which regulatory interactions take place. Rather, the graphical representation of CTBNs encodes possible relations of causality among variables.

In this work, CTBNs were applied to reconstruct the regulatory network that controls the TH17 cell differentiation in humans. We made use of an unevenly distributed time-course microarray experiment^22^, where human CD4^+^ T cells were isolated from umbilical cord blood and TH17 differentiation was initiated with polarizing cytokines. The inferred regulatory network predicted the gene *Prdm1,* whose role in human TH17 cells was previously unknown, as playing a key role in TH17 differentiation process. By perturbing the mRNA expression of *Prdm1*, we were able to confirm its role in regulating the TH17 differentiation process. In addition, the network highlighted the gene *Socs3* as being the second major hub node of the process. Interestingly, both genes are known to negatively regulate TH17 differentiation in murine tissues^23^,^24^. These findings suggests that negative regulators may exert a major control on TH17 differentiation process. This study represents the first *in vitro* biological validation of a regulatory network inferred using CTBNs as network reconstruction method.

## Network inference

**Figure 1:**
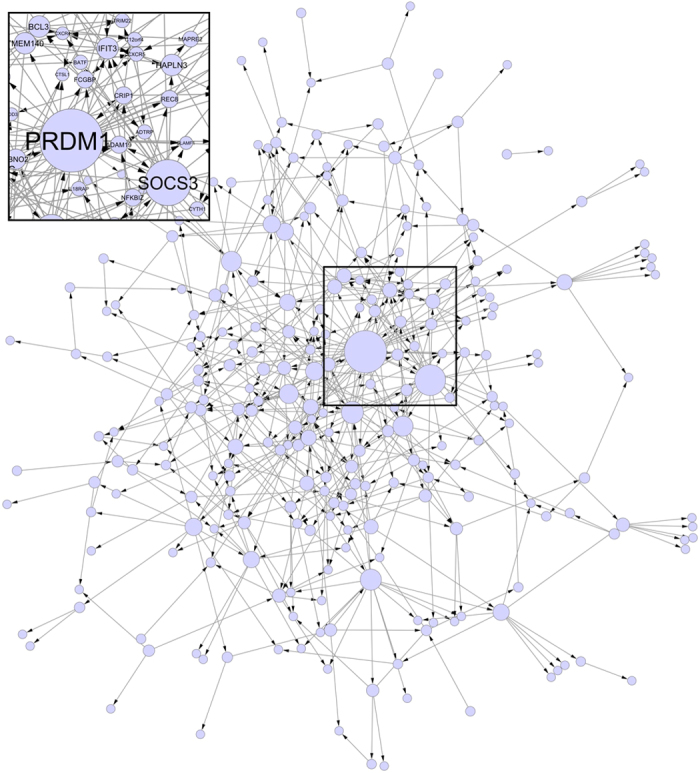
Inferred network of human TH17 cell differentiation. Node size is proportional to the number of outgoing arcs.

The microarray measurements used to learn the regulatory network were taken at 10 different time-points over the span of 72 h following the initiation of the differentiation process. As a control, the same time measurements were taken for the unstimulated cells (naïve CD4^+^ T). The experiment was repeated over three biological replicates (data available at the NCBI Gene Expression Omnibus with accession number GSE35103). The time measurements are unevenly distributed over the duration of the experiment; this makes the data particularly suitable for learning with CTBNs, which implement an explicit representation of the time. After pre-processing steps*, greedy* structural learning of CTBNs was applied (section Methods). The resulting inferred network (Fig. 1) had 258 nodes and 498 directed arcs. The major hub and root node resulted to be the one associated with the gene *Prdm1*, suggesting that this gene may have a marked influence on the TH17 differentiation process.

The *Prdm1* gene encodes the B lymphocyte-induced maturation protein 1. In mice it represents a transcriptional repressor that acts on terminal differentiation of B, T cells, and on TH1 and follicular TH cell subsets. PRDM1 has recently been defined as regulatory molecule for controlling effector and memory lymphocyte differentiation^25^. In B cells it acts by antagonistic suppression of the *c-myc*, *Bcl6* and *Pax5* genes^25^. It can also attenuate TH1 cells and control the development and suppressive function of regulatory T cells (Tregs). Indeed, mice lacking PRDM1 in T cells markedly increased TH1 and TH17 cells, and developed highly proliferative and activated lymphocytes^26^. The protective function of PRDM1 is also demonstrated in autoimmune encephalomyelitis (EAE)^27^, since mediated suppression of PRDM1 of TH1 and TH17 cells resulted in EAE protection in mice and suggest a PRDM1-targeted therapeutic strategy against encephalomyelitis. However, in murine T cells the role of PRDM1, which also appears not to be a trivial target to perturb^4^, is still under debate. Recently, a novel model of conditional deletion in peripheral cells of PRDM1 revealed a positive role of this molecule on TH17 differentiation^28^. This is in contrast with what emerged from previous studies, where the *Prdm1* gene was found to negatively regulate the secretion of IL-17 from murine TH17 cells^29^.

In the human case, the role of PRDM1 in TH17 cell differentiation remains unknown. The prediction derived from the inferred network of *Prdm1* being a major hub is consistent with what emerged from our previous study, where CTBNs were applied to the TH17 differentiation domain, but to a murine dataset^21^. Interestingly, in the murine case the gene *Prdm1* was predicted to play a role in balancing TH17 pathogenic and non-pathogenic cells: the inferred network highlighted a regulation loop between *Prdm1* and the gene *Tnfsf11* (alias *Rankl*), which is known to be a marker of pathogenic TH17 cells in inflammation^30^. The regulation loop is an indicator of a possible balancing mechanism between *Prdm1* and *Tnfsf11* genes. However, the predicted interactions were not experimentally validated for the murine dataset.

**Figure 2:**
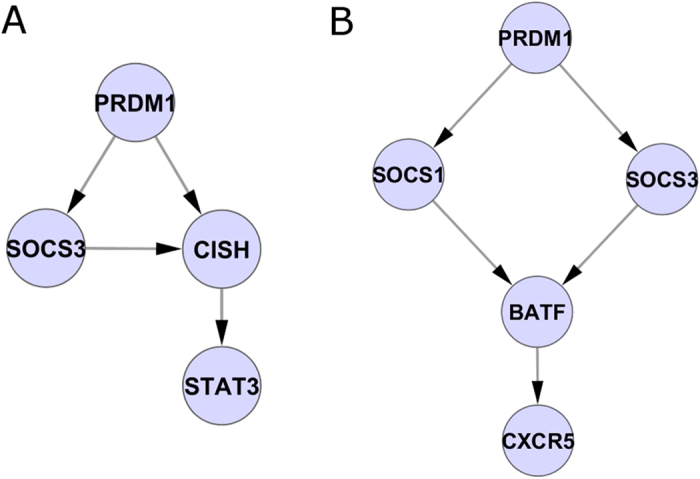
Specific regulation mechanisms from the inferred network. (**A**) Known pathway of negative regulation of SOCS3 on STAT3 (**B**) Regulatory interaction between PRDM1, SOCS3, SOCS1, BATF and CXCR5 as predicted by the inferred network.

The second major hub in the inferred network resulted to be the one associated with gene *SOCS3* (Fig. 1)*. SOCS3* is a suppressor of cytokine signaling, a negative feedback regulator of STAT3-activating cytokines^31^, that is known to be a major negative regulator of TH17 cells^32^. More interestingly, SOCS proteins are known as being involved in important mechanisms of negative regulation of the JAK-STAT pathway^33^. It has been shown that SOCS3 mediates inhibition of TH17 differentiation upon IL-23 or IL-6 exposure^34^ and in conditions of hyperactivation of STAT3. In addition, mesenchymal stem cells also inhibit TH17 cell differentiation through the activation of SOCS3^35^. Therefore, SOCS3 also plays a suppressive role in TH17 induction by negatively regulating STAT3 activation. This specific well-known mechanism emerged from the inferred network (Fig. 2A), where a direct interaction emerged between PRDM1, SOCS3, CISH and STAT3; CISH belongs to the suppressors of cytokine signaling (SOCS) family as well.

Other known key regulators of the TH17 differentiation process, such as BATF, STAT3, MAF, etc. are placed, together with a number of other genes, at an intermediate level of the inferred network hierarchy. This is consistent with these regulators not being known to act as early activators/repressors of the differentiation process.

Interestingly, the network revealed how the major hubs SOCS3 and PRDM1 directly control the transcription factor BATF (Fig. 2B), which is a key positive regulator of the TH17 differentiation program. The network predicted BATF to ultimately control the expression of CXCR5, characterizing the TH17 differentiated subset, as is already known in the literature^36^.

## Experimental validation

**Figure 3:**
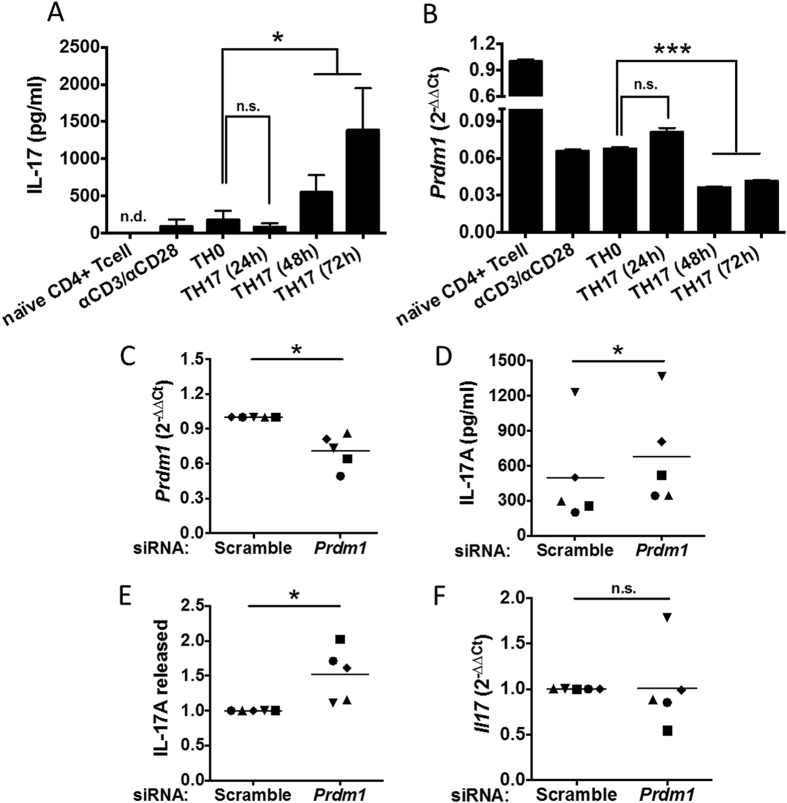
Experimental validation through Prdm1 perturbation. *Prdm1* perturbation boosts IL-17 differentiation program. IL-17A (**A**) and *Prdm1* mRNA levels (**B**) were assessed by ELISA and qPCR, respectively, in naïve CD4^+^ T cells and CD4^+^ T cells stimulated as follow: αCD3/αCD28, TH0 polarization for 72 h, or TH17 polarization for the indicated time. (**C**) qPCR was used to validate the siRNA-mediated reduction of *Prdm1* expression levels upon siRNA-mediated perturbation. Graphs show the mean of 5 independent experiments. (**D**,**F**) IL-17A cytokine release (**D**,**E**) and mRNA (**F**) from siRNA-treated CD4^+^ T cells following TH17 polarization (48 h) was measured by ELISA and pPCR, respectively. In panel E results are shown as relative values normalized to the corresponding scramble control. Significance was calculated using two-tailed paired t-test (**A**,**B**,**D**) and one-sample t-test (**C**,**E**,**F**). *P value < 0.05; ***P value <0.001; n.s., non significant.

**Figure 4:**
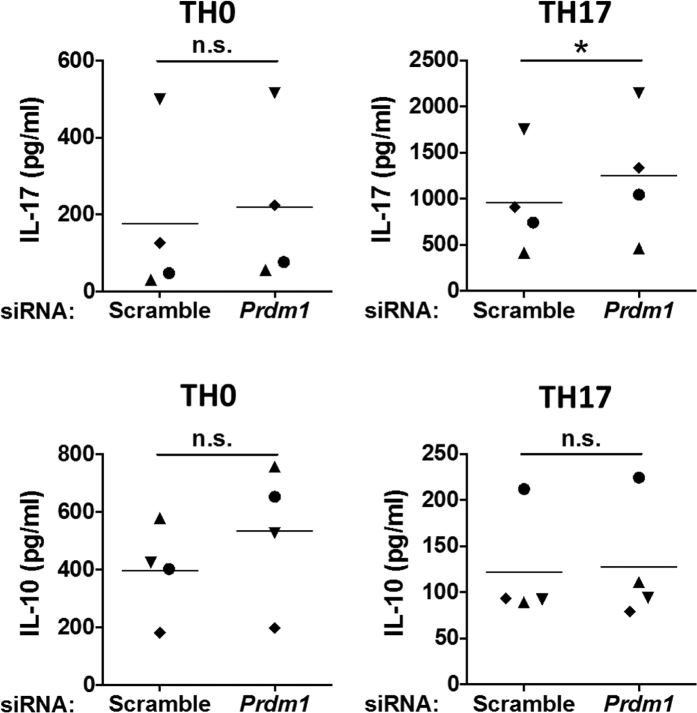
Effect of Prdm1 perturbation on IL-17A and IL-10 secretion. *Prdm1* perturbation increases IL-17A, but not IL-10, release. Secretion of IL-17A (upper) and IL-10 (lower) was measured in siRNA-treated CD4^+^T cells polarized towards TH0 (left) or TH17 (right) cells for 72 h. Two-tailed paired t-test has been performed. n.s., not significant; *P value < 0.05.

To validate the modulatory role of *Prdm1* gene in human TH17 cell differentiation, as predicted by the structural learning of CTBNs, we established a specific siRNA-mediated approach to perturb the expression of *Prdm1* mRNA in human CD4^+^ T cells during TH17 differentiation. Peripheral blood mononuclear cells were isolated by density gradient centrifugation obtained from anonymous blood healthy donors. CD4^+^ T cells were purified by negative selection and subsequently TH17 polarization was induced with IL-1β, IL-6 and TGF-β in the presence of the neutralizing anti-IFNγ and anti-IL-4 antibodies for 72 h. The conversion of naïve CD4^+^ T cells into differentiated TH17 cells was particularly relevant at 72 h, as indicated by high IL-17 production (Fig. 3A) and other peculiar cytokines characterizing TH17 differentiation (Supplementary Fig. 1). *Prdm1* expression was significantly reduced over time during TH17 differentiation (Fig. 3B). Following *Prdm1* perturbation, IL-17 protein levels significantly increased (Fig. 3C–E), indicating that *Prdm1* plays an inhibitory role in the human TH17 differentiation program. As expected, the regulation of *Il17a* mRNA was not significantly increased (Fig. 3F) as the gene control of PRDM1 on *Il17* gene requires higher levels of PRDM1 expression, which can be found on TH1 or TH2 cells as already shown in murine T cells^29^. Interestingly, while IL-17 release increased upon *Prdm1* perturbation, the production of IL-10, an anti-inflammatory cytokine released by TH17 cells, was unaffected (Fig. 4), suggesting a specific regulatory role of *Prdm1* in IL-17 secretion but not IL-10. In addition, unaffected was also the expression of other TH17 cytokines as *Il21*, *Il22* or *Rorc* (Supplementary Fig. 2).

## Conclusion

The exact role of *Prdm1* in human TH17 cells was unknown. Our study identifies human *Prdm1* as a major negative regulator of human TH17 differentiation acting in the early phase of the process. This is consistent with the observed functions of *Prdm1* in mouse studies. These findings provide important new insights to better identify new potential drug candidates that control TH17 polarization in autoimmune diseases. In addition, our results suggest that negative regulators may exert a major role in the initial phases of the process of TH17 differentiation. For the first time continuous time Bayesian networks have been used as hypothesis generation tool for wet-lab biological experiments, and have been confirmed as a valid method for inferring gene regulatory networks from time-course expression data.

## Methods

### Data preprocessing and learning parameters

Raw data was analyzed using R version 3.1.2 and the Bioconductor package. Data was log2 transformed and log2 normalized. Similarly to what was done in the original study^22^, probes with detection *P* values <0.05 were discarded, as well as those with a SD <0.15 over all the samples. Differentially expressed genes were calculated for each time-point. Genes that were differentially expressed (limma t-test <0.05, FDR corrected) and with a fold-change >1 or <−1 in at least one time-point were selected. The resulting dataset was composed of 284 genes. Prior to learning with CTBNs, fold-change data was discretized into 3 equal bins, with the first bin corresponding to fold-change values ≤−1, the second bin corresponding to values >−1 and <1, the third bin corresponding to values ≥1. Genes whose bin resulted to be constant across all time-points were discarded. For the structural learning of CTBNs, hyperparameters *α* and *τ* were set to 0.01 and 5, respectively, while the maximum number of parents allowed per node was set to 5. *Greedy hill-climbing* learning was run using the CTBN MATLAB Toolbox developed at the MAD (Models and Algorithms for Data and text mining) Lab at the University of Milano-Bicocca, Milan, Italy.

### Human CD4^+^ T cell isolation and culture

Peripheral blood mononuclear cells were isolated by Ficoll-Hypaque density gradient centrifugation of blood cones obtained from anonymous healthy donors (Blood Bank of Health Science Authority of Singapore; NUS-IRB 10–250). Naïve CD4^+^ T cells were purified by negative selection using CD4^+^ T Cell isolation kit (Miltenyi) following the manufacturer’s instructions. CD4^+^ T cells purity was assessed by flow cytometry using an APC-labeled anti-human CD4 antibody (clone OKT4, Biolegend) and was routinely >95%. Cells were activated as previously described^14^. Briefly, cells (0.25 × 10^6^) were activated with plate-bound anti-CD3 antibody (750 ng/24-well culture plate, Miltenyi) and soluble anti-CD28 antibody (1 μg/ml; Miltenyi) in *X-vivo* 15 serum-free medium (Lonza) supplemented with 100 U/ml penicillin, 100 μg/ml streptomycin and 2 mM of L-glutamine (all from Gibco). TH17 polarization was induced with recombinant human IL-1β (10 ng/ml), IL-6 (20 ng/ml) and TGF-β (10 ng/ml) in the presence of neutralizing anti-IFNγ antibody (1 μg/ml) and anti-IL-4 antibody (1 μg/ml; all from Miltenyi) for the indicated time. TH0 polarization was triggered adding neutralizing anti-IFNγ and anti-IL-4 antibodies without any additional cytokine.

### Small interfering RNA-mediated knockdown

CD4^+^ T cells (5 − 10 × 10^6^) were nucleofected with siRNA control (scramble) or specific siRNA targeting *Prdm1* (600 nM, Dharmacon) using Amaxa Nucleofector^TM^ Device (program U-014) following the manufacturer’s instructions (Lonza). After nucleofection, CD4^+^ T cells were incubated in *X-vivo* 15 complete medium for 6 h and, subsequently transferred in a 12-well plate pre-coated with anti-CD3 antibody (750 ng/24-well culture plate, Miltenyi) for 42 h before inducing TH17 differentiation. Total RNA was collected to test silencing efficacy by Q-PCR.

### Quantitative RT-PCR

RNA was isolated using the RNeasy method (Qiagen) and treated with DNase I (Promega) following the manufacturer’ instructions. Quantitative real-time PCR (Q-PCR) was performed in triplicate using GoTaq Q-PCR Master Mix (Promega) using the following validated SYBR Green primer pairs: *Prdm1*, forward 5′-AAAAGAAACATGACCGGCTACAAG-3′, reverse 5′- GGTGGACCTTCAGATTGGAGA-3′; *Il17*, forward 5′- TCTGTGATCTGGGAGGCAAAGTG-3′, reverse 5′- GAAGGAGTTGGGGCAGTGTGGAG-3′; *Il21*, forward 5′- CAGGGAGAAGACAGAAACACAGAC -3′, reverse 5′- TACCTTTTGGAGAAGTGATTTGAA -3′; *Il22*, forward 5′- AAGTGCTGTTCCCTCAATCTG-3′, reverse 5′- AGCTTTTGCACATTCCTCTGG -3′; *Rorc*, forward 5′- CGGCAGCGCTCCAACATCTT-3′, reverse 5′- GGCACACCGTTCCCACATCTC-3′ *GAPDH*, forward 5′- CCACATCGCTCAGACACCAT-3′, reverse 5′-GGCAAC AATATCCACTTTACCAGAGT-3′. Amplification was performed using the 7500 real-time PCR system (Applied Biosystems) and relative expression level of *Prdm1* was evaluated using the 2^-ΔΔCt^ method. Values were normalized for the expression of the housekeeping gene (*GAPDH*) and the Ct value of the naïve CD4^+^ T cells (Fig. 3B) or scramble siRNA control (Fig. 3C,F, Supplementary Fig. 2) was used as a calibrator.

### Cytokine secretion measurement

CD4^+^ T cells differentiation toward TH17 phenotype was evaluated as IL-17 A secretion by ELISA (Biologend). IL-10 release was measured by ELISA (Biolegend).

### Statistical analysis

Data were analyzed using Prism 6 software (GraphPad) and statistical significance was calculated using one-sample t-test or paired two-tailed t-test.

## Additional Information

**How to cite this article**: Acerbi, E. *et al*. Continuous time Bayesian networks identify Prdm1 as a negative regulator of TH17 cell differentiation in humans. *Sci. Rep.* **6**, 23128; doi: 10.1038/srep23128 (2016).

## Supplementary Material

Supplementary Information
