# Supplementary material for: Continuous time Bayesian networks identify Prdm1 as a negative regulator of TH17 cell differentiation in humans
Source: Sci Rep. 2016 Mar 15;6:23128. doi: 10.1038/srep23128 (PMC4791550; doi:10.1038/srep23128)
Supplement: Supplementary Information [file srep23128-s1.doc]

**Continuous time Bayesian networks identify Prdm1 as a negative regulator of TH17 cell differentiation in humans**

Enzo Acerbi^1,2,*,ƚ^, Elena Viganò^2,ƚ^, Michael Poidinger^2^, Alessandra Mortellaro^2^, Teresa Zelante^3,2,○^ and Fabio Stella^4,○^

^1^Singapore Centre on Environmental Life Sciences Engineering (Nanyang Technological University), Singapore 637551.

^2^Singapore Immunology Network (SIgN), A*STAR, 8A Biomedical Grove, Immunos #04-06, Singapore 138648.

^3^Department of Experimental Medicine, University of Perugia, 06132 Perugia, Italy.

^4^Department of Informatics, Systems and Communication, University of Milano-Bicocca, Viale Sarca 336, Building U14, 20126 Milan, Italy.

ƚ These authors contributed equally

◦ These authors contributed equally

* Correspondence to: eacerbi@ntu.edu.sg

**
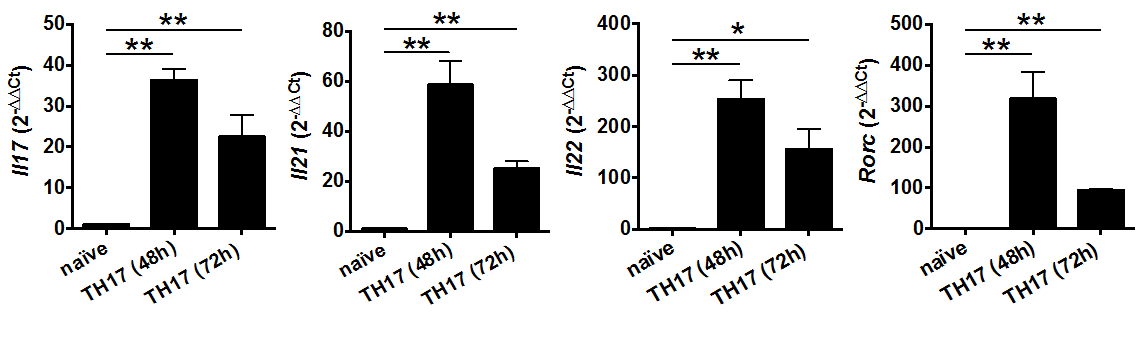
**

**Supplementary Figure 1. *Il17, Il21, Il22* and *RORc* expression during TH17 differentiation.** Expression of *Il17, Il21, Il22* and *RORc* was measured by quantitative PCR in naïve CD4^+^ T cells activated under condition that promote TH17 differentiation for the indicated time. One sample t-test has been performed.* P value < 0.05; ** P value < 0.001.


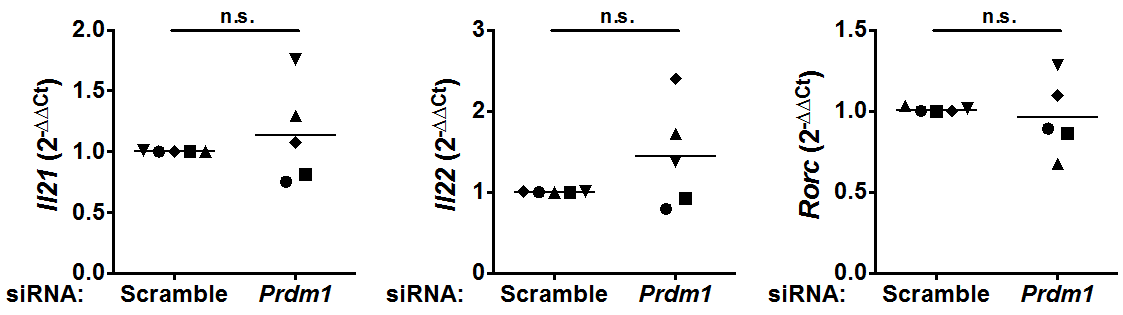


**Supplementary Figure 2**: **Effect of *Prdm1* perturbation on *Il21*, *Il22* and *Rorc* mRNA expression.** Reduction in expression of *Prdm1* does not affect *Il21*, *Il22* and *Rorc* mRNA expression at 48 hours. *Il21*, *Il22* and *Rorc* mRNA levels were assessed by qPCR in siRNA-treated CD4^+^ T cells following TH17 polarization (48h). Significance was calculated using one-sample t-test (C, E and F). n.s., non significant.
